# Behaviour Change Interventions in Healthcare

**DOI:** 10.3390/ijerph19127055

**Published:** 2022-06-09

**Authors:** Simão Pinho, Rute Sampaio

**Affiliations:** 1 Department of Biomedicine, Faculty of Medicine, University of Porto, 4200-319 Porto, Portugal; rutesampaio@med.up.pt; 2 São João Universitary Hospital Centre, Alameda Prof. Hernâni Monteiro, 4200-319 Porto, Portugal; 3 CINTESIS—Center for Research in Health Technologies and Services, R. Dr. Plácido da Costa, 4200-450 Porto, Portugal

## 1. Introduction

In September 2021, the Special Issue “Evidence-based Behaviour Change Interventions in Healthcare” was proposed as the manifestation of a will to compile multidisciplinary works of academic research focused on the effect of health education, psychology, and socio-cultural dimensions on the improvement in health habits in the population. Indeed, the Special Issue was devised as a step in answering the need for a holistic, yet robust and rigorous approach to the study of patients’ behaviour. The way behaviour can be shaped, and the impact that flexibility has on a subject’s health in particular, was deemed of great pertinence.

The promotion of behaviour changes, especially regarding health habits, is an infinitely interesting but challenging area of research. In fact, even with the best of intentions, it can be an ethically questionable practice, as one might argue it interferes with an individual’s free will and right to self-determination. As such, behaviour change interventions in health must carefully balance the benefits they may bring, be it to a population as a whole or the individual itself, with their personal right to guide their own behaviour. In many ways, an ideal behaviour change intervention is one that provides the individual with all the necessary tools to make the decision that is best for them but ultimately allows them to make their own choice. Because of this, there is an optimism inherently tied to an ethical behaviour intervention; one must believe an educated person will make the healthiest choice. Interestingly, this notion is in line with Socrates’ view, asserted in the Protagoras, that no person does evil except out of ignorance [1]. 

## 2. Health Literacy

Health literacy is a multidimensional concept, found at the interplay between health practice, education, and public resources. One of the most recent interpretations of health literacy, found in the fifth iteration of the US Department of Health and Human Services’ Healthy People initiative, separates it into personal health literacy and organisational health literacy, highlighting the role of public and private organisations in informing patients’ decisions [2]. 

Besides promoting and perpetuating socioeconomic differences in the population, the lack of health literacy is also crucial in creating health disparities. In fact, it is one of the clearest reasons in explaining why poorer and less educated population groups have worse health outcomes [3].

In recent years, the impact of health literacy on public health has become increasingly evident. Indeed, the global burden of chronic diseases has greatly increased, in line with societal epidemiological transitions, and the importance of preventive healthcare has never been greater [4]. Compounding this panorama is the health literacy issues that the COVID-19 pandemic has brought to the spotlight: health misinformation is rampant, and, in a world where information is very easily and rapidly available, complex problems arise when people are met with contradictory statements. Often, reliable sources are silenced in a sea of misinformation driven by economic or political interests [5]. Because of this, there has been a global effort to rethink the role of health literacy, recognising it as a foundational principle in line with the objective of improving population health and well-being. 

## 3. Evidence-Based Education

When tackling health illiteracy, population education is naturally thought of as the best solution. Nevertheless, behind what might seem like an obvious response to the problem hides a very complex issue. It is easy to mistakenly equate educational interventions to the mere act of providing information. This, however, is simply not enough to improve health literacy. Information availability is but one obstacle, and simply making information easier to access for a population is not enough to increase its health literacy [6]. 

Consequently, successful, evidence-based educational interventions in health should be designed using a well-established psychological, behavioural framework, or, at least, come up with a new model that is used to guide the intervention. Having a theoretical framework allows researchers and stakeholders to devise deliberate, thought-out interventions [7]. This is important not only to ensure there is coherency in the process, but also to guarantee that an intervention is not only replicable, but also able to be adapted and improved in the future while maintaining the same rational approach. Unfortunately, there is a significant amount of published educational interventions that do not adhere to this philosophy. Such works add very little to the effort to find effective ways to improve public health and health literacy. Indeed, even if these interventions are found to be successful, they are extremely hard, if not outright impossible, to replicate, and it is difficult to interpret their success if there was no underlying theoretical framework.

In conclusion, there is a need for greater accuracy when developing and implementing behaviour change interventions in health. The intent behind the Special Issue “Evidence-based Behaviour Change Interventions in Healthcare” is to highlight this and promote a dialogue on the subject of using education to improve public health.
